# The Effects of Music Intervention on Pallidum-DMN Circuit of Schizophrenia

**DOI:** 10.1155/2020/4107065

**Published:** 2020-09-19

**Authors:** Yutong Yao, Hui He, Mingjun Duan, Shicai Li, Cheng Li, Xi Chen, Gang Yao, Xin Chang, Haifeng Shu, Hongming Wang, Cheng Luo

**Affiliations:** The Clinical Hospital of Chengdu Brain Science Institute, MOE Key Lab for Neuroinformation, University of Electronic Science and Technology of China, Chengdu 610054, China

## Abstract

Music intervention has been applied to improve symptoms of schizophrenic subjects as a complementary treatment in medicine. Although the psychiatric symptoms, especially for motivation and emotion, could be increased in schizophrenia, the underlying neural mechanisms remain poorly understood. We employed a longitudinal study to measure the alteration of striatum functional networks in schizophrenic subjects undergoing Mozart music listening using resting-state functional magnetic resonance imaging (fMRI). Forty-five schizophrenic inpatients were recruited and randomly assigned to two groups. Under the standard care with antipsychotic medication, one group received music intervention for 1 month and the other group is set as control. Both schizophrenic groups were compared to healthy subjects. Resting-state fMRI was acquired from schizophrenic subjects at baseline and after one-month music intervention and from healthy subjects at baseline. Striatum network was assessed through seed-based static and dynamic functional connectivity (FC) analyses. After music intervention, increased static FC was observed between pallidum and ventral hippocampus in schizophrenic subjects. Increased dynamic FCs were also found between pallidus and subregions of default mode network (DMN), including cerebellum crus and posterior cingulate cortex. Moreover, static pallidus-hippocampus FC increment was positively correlated with the improvement of negative symptoms in schizophrenic subjects. Together, these findings provided evidence that music intervention might have an effect on the FC of the striatum-DMN circuit and might be related to the remission of symptoms of schizophrenia.

## 1. Introduction

Schizophrenia is a complex psychiatric illness, which is always characterized by positive and negative symptoms and cognitive impairments [1], affecting about one percentage of the population worldwide [2]. Antipsychotic drugs are commonly chosen for prolonged treatments for patients with schizophrenia. The complementary therapy, such as music intervention, is also used for schizophrenia. Importantly, several studies indicated that music intervention could improve schizophrenic subjects' psychiatric symptoms [3]. Understanding this neurophysiological mechanism could gain a better understanding of complementary therapy and might improve further therapies on schizophrenia.

Music intervention, such as music listening or performance, is considered as a profound capacity to improve individuals' emotions and change our physiological behavior and subjective perception [4]. Furthermore, Glicksohn and Cohen found Mozart music listening could facilitate cognitive task performance of schizophrenic subjects [5]. Importantly, clinical reports revealed that music intervention could enhance the motivation and relationship-building qualities of schizophrenic subjects [6, 7], which are aberrant in patients with schizophrenia and further considered as negative symptoms of schizophrenia [8]. Previous studies have indicated that music intervention might improve schizophrenic subjects' positive symptoms through normalizing the salience, sensorimotor, and visual networks [9, 10], while there are few research studies investigating the relationship between improved negative symptom and altered functional feature within the brain network of schizophrenic subjects. One prominent theory documented that schizophrenic subjects are associated with increased subcortical dopamine [11]. The positive symptoms of schizophrenia could be related to a hyperactive subcortical dopaminergic system [11]. Excellent research has also revealed that abnormality in dopamine-mediated striatum system may be related to multiple deficient reward processing, which contributes to the negative symptoms of schizophrenic subjects [12]. The striatum is differentiated into caudate, putamen, and nucleus accumbens [13]. These subregions of striatum receive and send back information with the cortex. Furthermore, Blood and Zatorre indicated that ventral striatum would respond to music stimuli [14]. Thus, the striatum system might be an important region related to altered total symptoms through music intervention in schizophrenic subjects.

Resting-state functional magnetic resonance imaging (fMRI) has been employed in investigating the pathophysiology of schizophrenia [15–20]. Deficient static functional connectivity (FC) has been found in several functional networks of schizophrenia, respectively, such as default mode network (DMN), sensorimotor network, and salience network [17, 21–25]. Therefore, the symptom of schizophrenic subjects is referred to as a “dysconnection” syndrome, where the neuropathology arises from the altered functional interaction across brain regions [19, 26, 27]. Recent excellent research studies also documented that FC within the human brain is not stationary and changes over time [28, 29]. Moreover, the results of neuroimage study support and expand current knowledge about the pathophysiology of schizophrenia through dynamic FC [30]. Altogether, these perspectives posit that static and dynamic FC could provide complementary information about the pathophysiology of schizophrenia, while there is no study that explores the music intervention on the functional network of schizophrenia through static and dynamic FC analyses during recent years.

Therefore, in this study, static and dynamic FC analyses were used to investigate the effect of music intervention on striatum functional network of schizophrenia. Based on the findings from previous neuroimage studies, we hypothesized that the music intervention would positively improve the symptoms of schizophrenic subjects through FC changes between the regions of striatum and cortical cortex. We posit that these reflected FC changes are an important driving factor in pathological functional changes through music intervention on schizophrenia.

## 2. Materials and Methods

### 2.1. Demographic Characteristics

Forty-five schizophrenic subjects were recruited in this study from the clinical hospital of Chengdu Brain Science Institute (CBSI). The inclusion criterion for the inpatients was a diagnosis of schizophrenia based on the Structured Clinical Interview for the DSM-IV Axis I disorders—clinical version (SCID-I-CV). The exclusion criteria were acute psychotic symptoms, a secondary diagnosis of organic psychosis or dementia, and unstable drug treatment. The psychiatric symptoms of the patients were measured using Positive and Negative Symptom Scale (PANSS). A set of matched MRI data of healthy controls (HC, *n* = 19) were obtained from CBSI research databases, which were screened for a history of medical or neuropsychiatric illness, as well as for major neurological or psychiatric illness in their first-degree relatives. The study was approved by the Ethics Committee of the clinical hospital of CBSI in accordance with the Helsinki Declaration. These data have been used to measure the music intervention on static global FC and insular networks, respectively, in schizophrenic subjects [9, 10]. In this study, we focused on music effects on static and dynamic FC of striatum in schizophrenia.

### 2.2. Design and Content of Music Intervention

A quasirandomized controlled trial was performed in this study. The experimental schizophrenic patients and controlled patients were compared using the reference of the HCs. Then, the patients were randomly divided into two groups by psychiatrists. Twenty-two schizophrenic subjects were selected as the music intervention group (MTSZ). Twenty-three inpatients were chosen as controls (UMTSZ). One professional music therapist participated in this study. Two groups of schizophrenia are chronic schizophrenic subjects with a stable drug treatment strategy (changeless antipsychotic drugs and their doses) in CBSI. Additionally, MTSZ inpatients listened Mozart's sonata K. 448 (half an hour per day, 30 days), which has been widely used in scientific studies to assess the effects of music [31, 32]. During each session, the inpatients were peaceful listening to the music of Mozart's sonata K. 448 from a stereo system in a quiet room.

### 2.3. Data Acquisition and Image Preprocessing

Imaging was conducted on a 3 T MRI scanner (GE DISCOVERY MR750). During scanning, we used foam padding and earplugs to reduce head motion and scanner noise, respectively. High-resolution T1-weighted images were acquired using a 3-dimensional fast spoiled gradient-echo sequence (repetition time (TR) = 6.008 ms, flip angle (FA) = 9°, matrix = 256 × 256, field of view (FOV) = 256 × 256 mm^2^, slice thickness = 1 mm, no gap, and 152 slices). Subsequently, resting-state functional MRI data were acquired using gradient-echo echo-planar imaging sequences (TR = 2000 ms, echo time (TE) = 30 ms, FA = 90°, matrix = 64 × 64, FOV = 240 × 240 mm^2^, slice thickness/gap = 4 mm/0.4 mm, and number of slices = 35), with an eight-channel phased-array head coil. All subjects underwent a 510-second resting-state scan to yield 255 volumes. During resting-state fMRI, all subjects were instructed to have their eyes closed and to move as little as possible without falling asleep.

Functional image preprocessing was performed using NIT (Neuroscience Information Toolbox) [33] according to a standard pipeline and briefly described here. Slice time and head motion correction and normalization (3 mm *∗* 3 mm *∗* 3 mm) into EPI template were performed. Further, the images were spatial smoothed (FWHM 6 mm). Detrending analysis was performed. Temporal filtering was performed at bandpass 0.01–0.08 Hz. Then, the sources of nuisance signals were removed from these images, including six motion parameters and their first temporal derivative, white matter signal, and cerebrospinal fluid signal. The global signal was not regressed out [34]. Power reported that head motion has a substantial impact on the functional map [35]. Thus, in this study, subjects who had a maximum translation in any of orthogonal direction larger than 3 mm or rotation larger than 3 degrees were excluded. Moreover, framewise displacement (FD) was evaluated for all subjects as suggested by Power et al.

Structural images were processed using SPM8 toolbox. The structural image was spatially normalized to MNI space using a diffeomorphic anatomical registration through exponentiated lie algebra (DARTEL) and segmented into gray matter (GM), white matter, and cerebrospinal fluid. Then the segmented GM was modulated using nonlinear deformation. The normalized GM score was calculated as follows: GM score divided by the total intracranial volume. The normalized GM score of subjects was as a variable in the statistical analysis to correct for the global GM volume of different subjects.

### 2.4. Static and Sliding Window-Based Dynamic Functional Connectivity Analyses

First, we calculated static functional networks of the striatum system. Six subregions within striatum systems were defined based on the automated anatomical labeling (AAL) template, including left and right caudate, left and right putamen, and left and right pallidum. The mean BOLD time courses of these regions were extracted. Subsequently, the Pearson correlation coefficients were calculated between these regions and all voxels in the brain. This correlation coefficient was considered as static FC based on the whole time course. The resulting values were transformed through Fisher's *r*-to-*z* transformation.

We calculated the sliding window-based dynamic functional map for each subregion of the striatum system as follows: first, the time course was segmented into shorter windows. In the sliding window-based dynamic functional connectivity analysis, the optimal window length is an open area of research. Recent study has indicated that the minimum window length should be no less than 1/*f*_min_ [36]. Thus, the time courses were segmented into 100 s (50TR) windows (*f*_min_ = 0.01 Hz), sliding the onset by 2 s (1TR). Second, within each window, the Pearson correlation analysis was performed between the subregions of striatum and all voxels, respectively. Finally, the dynamic FC was estimated by calculating the standard deviation (SD) in correlation coefficients at each voxel.

### 2.5. Statistical Analysis

First, to measure the effects of music intervention on static and dynamic functional networks, the statistical analysis was performed on the longitudinal date of MTSZ and UMTSZ groups through repeated-measure ANOVA. The repeated-measure ANOVA was performed with age, gender, education years, GM, and medication dosage as covariates. Multiple comparisons correction was performed using a high threshold (*p*=0.001) and an extent threshold based on Gaussian random field theory (*p*_corrected_=0.05). For the altered regions, we extracted the FC value in MTSZ and UMTSZ groups for post hoc analysis.

Second, for the altered FC from repeated-measure ANOVA, we established the baseline abnormalities between HC and MTSZ, and UMTSZ, respectively. Two-sample *t*-test was calculated between HC and schizophrenic subjects (MTSZ and UMTSZ of baseline). Using the different FC between HC and patients as a reference, the inverse FC (1 month minus baseline) might be defined as normalized FC through music intervention in MTSZ.

### 2.6. Correlations with Pathological Factors

The partial correlation analysis was performed between the mean changed static/dynamic FC (1 month later minus baseline) with altered symptom scores (1 month later minus baseline) of schizophrenic subjects with age, gender, illness duration, education years, GM, and medication dosage as covariates in this study.

## 3. Results

### 3.1. Demographic and Clinical Data

Nine subjects were excluded because of excessive head motion. Thus, 18 MTSZ, 18 UMTSZ, and 19 HC subjects were included in the following analysis (Table 1). There is no significant difference between MTSZ and UMTSZ groups in terms of age, gender, education years, duration of illness, and medication dosage in chlorpromazine (CPZ). The healthy and schizophrenic groups were also matched for age, gender, and education years. Details of demographic characteristics of MTSZ, UMTSZ, and HC groups are shown in Table 1. Moreover, main effect of music intervention and music intervention *∗* time interaction on symptoms was observed in the PANSS (Table 2).

### 3.2. Music Intervention Effects on Static and Dynamic Functional Connectivity

**Figure 1:**
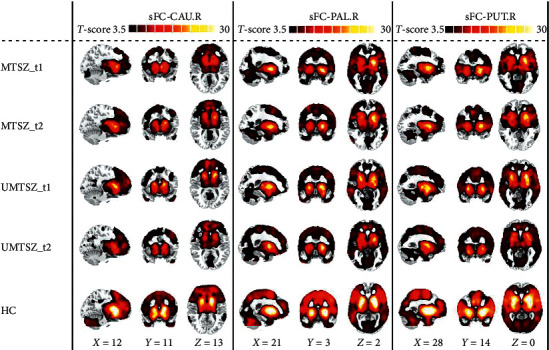
One sample *t*-test maps of each group. MTSZ_t1: music intervention schizophrenia group at baseline; MTSZ_t2: music intervention schizophrenia group at one month; UMTSZ_t1: nonmusic intervention schizophrenia group at baseline; UMTSZ_t2: nonmusic intervention schizophrenia group at one month; HC: healthy control; sFC: static functional connectivity; CAU.R: right caudate; PAL.R: right pallidum; PUT.R: right putamen.

**Figure 2:**
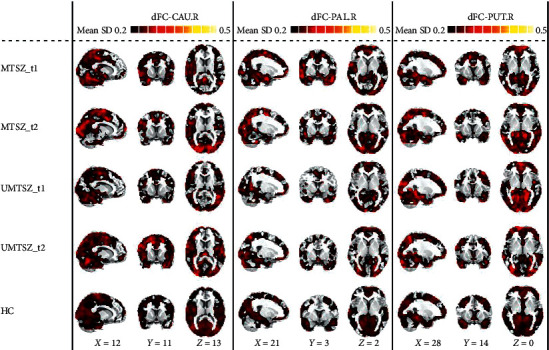
Mean dynamic functional maps of each group. MTSZ_t1: music intervention schizophrenia group at baseline; MTSZ_t2: music intervention schizophrenia group at one month; UMTSZ_t1: nonmusic intervention schizophrenia group at baseline; UMTSZ_t2: nonmusic intervention schizophrenia group at one month; HC: healthy control; dFC: dynamic functional connectivity; SD: standard deviation; CAU.R: right caudate; PAL.R: right pallidum; PUT.R: right putamen.

**Figure 3:**
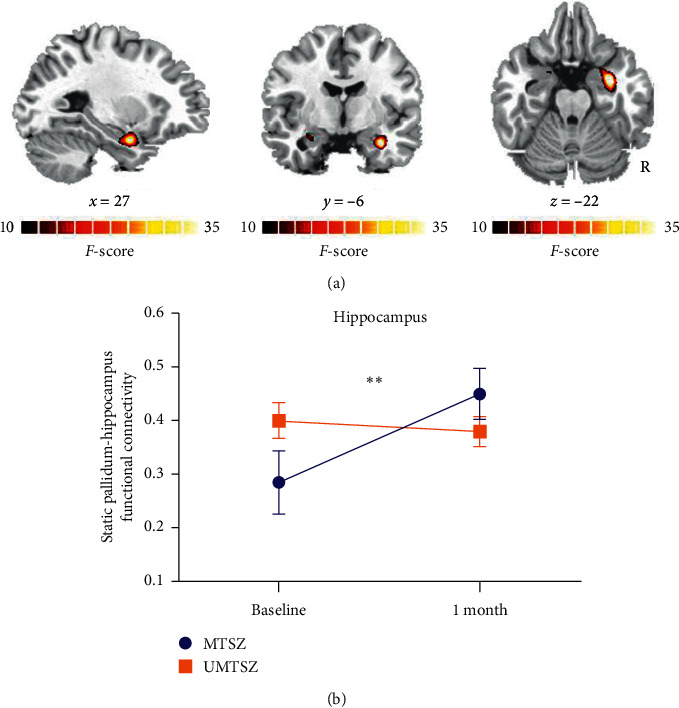
Music intervention on static FC. (a) Significant music intervention *∗* time interaction on static FC between right pallidum and left hippocampus through repeated-measure ANOVA. (b) The post hoc results (mean value ± standard deviation), ^*∗∗*^*p* < 0.01, significantly increased static functional connectivity in MTSZ.

**Figure 4:**
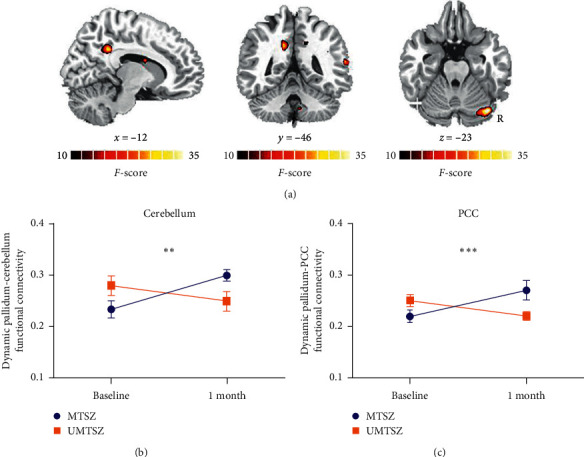
Music intervention on dynamic FC. (a) Significant music intervention *∗* time interaction on dynamic FC between right pallidum and cerebellum and posterior cingulate cortex (PCC) through repeated-measure ANOVA. (b, c) The post hoc results (mean value ± standard deviation), ^*∗∗*^*p* < 0.01, ^*∗∗∗*^*p* < 0.001, significantly increased static functional connectivity in MTSZ.

Figures 1 and 2 display the spatial pattern of static and dynamic FC of each subregion within striatum. First, significant music intervention *∗* time interaction effect was observed on static FC between right pallidum and left hippocampus (Figure 3(a) and Table 3). Post hoc analysis found that there was no difference between MTSZ and UMTSZ at baseline. Increased static FC was observed in MTSZ after music intervention compared to the baseline, while there was no change in UMTSZ (Figure 3(b) and Table 4). Second, regions displayed music intervention *∗* time interaction effects were found on dynamic FC between right pallidum and several subregions within DMN, including cerebellum crus and posterior cingulate cortex (PCC) (Figure 4(a) and Table 3), with increase in MTSZ following music intervention and no significant change in UMTSZ group (Figures 4(b) and 4(c) and Table 4). Furthermore, we found decreased dynamic pallidum-PCC FC between HC and MTSZ, and UMTSZ, respectively (Table 5).

**Figure 5:**
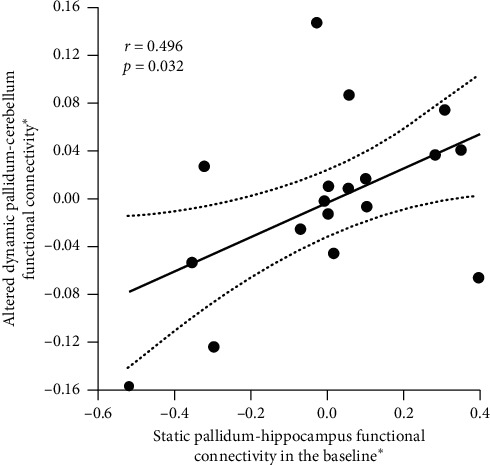
The relationship between static pallidum-hippocampus FC at baseline and altered dynamic pallidum-cerebellum FC in the MTSZ group. ^*∗*^Residual value after the regression analysis.

Another post hoc analysis was performed to determine the relationship between static FC and dynamic FC of striatum network in schizophrenic subjects. In MTSZ, we found a positive relationship (*r* = −0.496 and *p*=0.032; Figure 5) between static left pallidum-left hippocampus FC of baseline and altered dynamic left pallidum-cerebellum crus FC. However, this correlation was not observed in UMTS (*r* = 0.079 and *p*=0.627).

### 3.3. Relationship between Altered FC and Change Scores of PANSS

**Figure 6:**
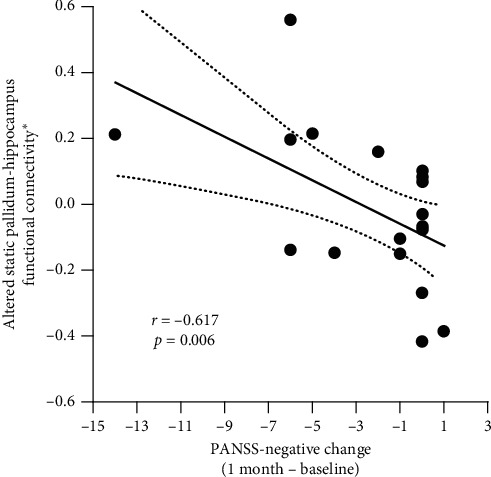
The relationship between altered static functional connectivity score and change score (1 month minus baseline) of negative symptom in the MTSZ group. ^*∗*^Residual value after the regression analysis.

Altered pallidum-hippocampus static FC showed a significant negative correlation with the change score of negative score (*r* = −0.617 and *p* = 0.006) of PANSS in the MTSZ group. To clearly show this relationship, the value of FC in Figure 6 regressed out the controls value through general linear model, including age, gender, illness duration, education years, GM, and medication dosage. There is no significant relationship between altered FC and altered positive symptoms of MTSZ subjects. Moreover, no significant correlation was observed between altered static or dynamic FC with the FD score, as well as medication dosage in the MTSZ group.

## 4. Discussion

This study explored the effects of music intervention on static and dynamic striatum functional networks in schizophrenic subjects alongside the use of antipsychotic therapy. After listening to Mozart's sonata music, we found schizophrenic subjects appeared to increase static FC between pallidum and ventral hippocampus and enhance dynamic FC between pallidum and cerebellum curs and PCC. All these altered functional regions have been documented which are previously identified as key regions of DMN [37]. Moreover, psychiatric symptom analysis found that altered static pallidum-hippocampus FC was negatively related with the changes of negative symptom in MTSZ subjects. Importantly, decreased dynamic pallidum-PCC FC related to HC could be improved through music intervention. Thus, altered dynamic pallidum-PCC FC might be defined as normalized FC through music intervention in MTSZ. The increased striatum-DMN FC might be the target of music intervention and contribute to the improved symptoms of schizophrenia.

For decades, the dopamine hypothesis is the dominant theory to interpret the symptoms of schizophrenia [38]. Furthermore, Grace indicated that the pathophysiology of schizophrenia may be related to abnormal regulative processes for the dopamine system by other brain regions [39]. Studies in humans and animals have revealed converging lines of evidence highlighting the hippocampus is a central component, which contributes to the regulation of dopamine neuron responsivity [40–43]. Specifically, the activity of hippocampus drives the nucleus accumbens inhibition of pallidum to release the dopamine [40]. Blood and Zatorre indicated that ventral striatum (e.g., pallidum) would respond to music stimuli [14]. In this study, we found music intervention could increase static FC between pallidum and hippocampus in schizophrenic subjects. Based on these dysfunction hypotheses of dopamine system, our findings might demonstrate that music intervention might improve the modulatory processes for dopamine network and contribute to the improvement of symptoms of schizophrenia through increased static pallidum-hippocampus FC.

Hippocampus is one key region within DMN of the human brain. Hippocampal lesions would lead to FC alterations in DMN of neurological patients [44]. The electrophysiological signals of the hippocampus could also trigger distributed subregion activity within DMN of monkey, but not other cortical networks [45]. Importantly, recent excellent study has indicated that structure connectivity between bilateral hippocampus and FC of hippocampus-neocortex is important for information flow within DMN [46]. Moreover, subregions of DMN were possible hubs of interplay between distributed major functional networks contributing to advanced cognitive processes [46, 47]. Thus, the effects on hippocampus will likely lead to an altered FC of DMN, further improving the cognitive function. Specifically, several studies have reported that disruptions of DMN may be related to psychiatric symptoms and cognitive deficits in schizophrenia [48–50]. In this study, after music intervention, schizophrenic subjects showed increased dynamic FC between pallidum and subregions of DMN. Moreover, we found a positive relationship between static pallidum-hippocampus FC of baseline and altered dynamic pallidum-cerebellum FC in the MTSZ group. Based on the mentioned studies, our findings might reveal that altered static pallidum-hippocampus FC through music intervention might contribute to increased dynamic pallidum-DMN FC in schizophrenic subjects. These altered dynamic FC might further improve information flow among brain networks, which may contribute to the improvement of symptoms of schizophrenia. Moreover, significant relationship was observed between altered static pallidum-hippocampus FC and changes of negative symptom in the MTSZ group. For this relationship, we suggest that increased pallidum-hippocampus FC might contribute to the improved negative symptom of schizophrenia. Although the function of striatum is related to both positive and negative symptoms, the positive symptom-altered FC relationship should be extended in further study.

Several limitation issues need to be considered when interpreting the results of this study. First, all inpatients we chose are medically treated chronic schizophrenic subjects. We should validate our findings in first-episode schizophrenic subjects. Second, we only used Mozart's sonata K. 448 listening per day to measure the music intervention on schizophrenia. To avoid the specific effects of music intervention, further studies should add the experimental group that listens to favorite or random music to deal with effects of regular intervention. Moreover, further studies should assess the effects of other types of music on inpatients of schizophrenia and assess the relationship between altered FC and symptoms of schizophrenic subjects. Finally, more data of schizophrenic subjects might argue for the inconsistency between nonsignificant altered negative symptom and significant altered negative symptom-altered FC relationship. Thus, we should recruit more schizophrenic subjects to assess and validate the effects of music intervention on positive and negative symptoms in further study. These results might support and expand current knowledge about the underlying neural mechanisms of music intervention on schizophrenia.

## 5. Conclusion

By combining resting-state static and dynamic FC approaches, our results reveal that music intervention might improve prominent static and dynamic FC between pallidum and subregions of DMN in schizophrenic subjects. These findings provided new evidence that one important neural mechanism of music intervention might be associated with the striatum-DMN functional circuit and related to the remission of symptoms of schizophrenic subjects.

## Figures and Tables

**Table 1:** Participant fundamental information.

	MTSZ	UMTSZ	HC	*p*
Gender (male/female)	5/13	5/13	7/12	0.787^a^
Age (years)	45.38 ± 9.69	45.72 ± 7.63	44.42 ± 4.70	0.863^b^
Education level (years)	11.94 ± 3.24	11.22 ± 2.90	11.36 ± 2.81	0.641^b^
Duration of illness (years)	19.66 ± 11.11	18.00 ± 8.18		0.611^c^
Medication dosage in CPZ equivalents (mg)	339.23 ± 94.15	320.53 ± 142.50		0.645^c^
FD	Baseline: 0.04 ± 0.02	Baseline: 0.06 ± 0.04	0.05 ± 0.03	
	1 month: 0.05 ± 0.03	1 month: 0.06 ± 0.05		

MTSZ: music intervention schizophrenia; UMTSZ: nonmusic intervention schizophrenia; HC: healthy control; CPZ: chlorpromazine; PANSS: Positive and Negative Symptom Scale; FD: framewise displacement. Indicated values are shown as mean ± standard deviation; ^a^*p* values for the comparisons (chi-square test) among MTSZ, UMTSZ, and HC; ^b^*p* values for the comparisons (analysis of variance) among MTSZ, UMTSZ, and HC; ^c^*p* values for the comparisons (two-sample *t*-tests) between MTSZ and UMTSZ.

**Table 2:** The music intervention *∗* time interaction on PANSS in schizophrenic subjects through repeated-measure ANOVA.

	MTSZ	UMTSZ	MTSZ	UMTSZ	Interaction effects		Post hoc (paired *t*-test)	
	Baseline	Baseline	1 month	1 month	*F*_(d*f*)_	*p*	*p*	*p*
	18/18	18/18	18/18	18/18			(MTSZ)	(UMTSZ)
PANSS-total score	62.89 ± 17.41	64.11 ± 11.73	54.78 ± 14.56	63.50 ± 12.21	9.509_(1,35)_	0.004^*∗∗*^	0.002^*∗∗*^	0.513
PANSS-positive score	12.89 ± 4.38	10.67 ± 4.60	10.66 ± 3.3	10.61 ± 4.64	13.294_(1,35)_	0.001^*∗∗*^	0.002^*∗∗*^	0.331
PANSS-negative score	21.78 ± 9.24	23.39 ± 5.98	19.39 ± 8.88	23.11 ± 6.67	3.005_(1,35)_	0.092	0.017^*∗*^	0.738
PANSS-general score	28.22 ± 7.03	30.05 ± 5.97	24.72 ± 5.00	29.78 ± 6.05	8.228_(1,35)_	0.007^*∗∗*^	0.005^*∗∗*^	0.311

Indicated values are shown as mean ± standard deviation. PANSS: Positive and Negative Symptom Scale; ^*∗*^*p* < 0.05 and ^*∗∗*^*p* < 0.01.

**Table 3:** Significant music intervention *∗* time interaction on static and dynamic FC of pallidum.

Regions	MNI coordinates			*F*-score_(d*f*)_; *P*-score	Cluster voxels
	*x*	*y*	*z*		
Static FC					
HP.R	33	−5	−22	*F* = 29.03_(1,35)_; *P* = 4.952 × 10^−6^^*∗∗∗*^	39
Dynamic FC					
CERE.R	39	−73	−23	*F* = 30.69_(1,35)_; *P* = 3.122 × 10^−6^^*∗∗∗*^	52
PCC	−6	−45	32	*F* = 21.29_(1,35)_; *P* = 5.116 × 10^−5^^*∗∗∗*^	33

FC: functional connectivity; HP.R: right hippocampus; CERE.R: right cerebellum crus I; PCC: posterior cingulate cortex. ^*∗∗∗*^*p* < 0.001.

**Table 4:** The post hoc results of music intervention *∗* time interaction on static and dynamic FC of pallidum.

Regions	MTSZ (1 month minus baseline)	UMTSZ (1 month minus baseline)	MTSZ minus UMTSZ (baseline)	MTSZ minus UMTSZ (1 month)
	*T*-score_(d*f*)_; *P*-score	*T*-score_(d*f*)_; *P*-score	*T*-score_(d*f*)_; *P*-score	*T*-score_(d*f*)_; *P*-score
Static FC				
HP.R	*T* = 2.589_(35)_; *P* = 0.019^*∗*^	*T* = 0.399_(35)_; *P* = 0.694	*T* = −1.890_(34)_; *P* = 0.067	*T* = 0.073_(34)_; *P* = 0.942
Dynamic FC				
CERE.R	*T* = 3.514_(35)_; *P* = 0.002^*∗∗*^	*T* = −0.783_(35)_; *P* = 0.44	*T* = −1.868_(34)_; *P* = 0.070	*T* = 1.792_(34)_; *P* = 0.082
PCC	*T* = 4.298_(35)_; *P* = 5 × 10^−4^^*∗∗∗*^	*T* = −2.232_(35)_; *P* = 0.039^*∗*^	*T* = −1.969_(34)_; *P* = 0.057	*T* = 4.011_(34)_; *P* = 3 × 10^−4^^*∗∗∗*^

FC: functional connectivity; HP.R: right hippocampus; CERE.R: right cerebellum crus I; PCC: posterior cingulate cortex. ^*∗*^*p* < 0.05 and ^*∗∗*^*p* < 0.01.

**Table 5:** The comparison results of static and dynamic FC of pallidum network between HC and MTSZ, and UMTSZ at baseline.

Regions	MTSZ (baseline) minus HC	UMTSZ (baseline) minus HC
	*T*-score_(d*f*)_; *P*-score	*T*-score_(d*f*)_; *P*-score
Static FC		
HP.R	*T* = −2.101_(35)_; *P* = 0.042^*∗*^	*T* = −0.717_(35)_; *P* = 0.477
Dynamic FC		
CERE.R	*T* = −0.342_(35)_; *P* = 0.734	*T* = 1.503_(35)_; *P* = 0.141
PCC	*T* = −4.034_(35)_; *P* = 3 × 10^−4^^*∗∗∗*^	*T* = −2.297_(35)_; *P* = 0.027^*∗*^

HC: healthy control; FC: functional connectivity; HP.R: right hippocampus; CERE.R: right cerebellum crus I; PCC: posterior cingulate cortex. ^*∗*^*p* < 0.05 and ^*∗∗*^*p* < 0.01.

## Data Availability

The data used to support the results of this study are available from the corresponding author upon request.
